# Journal ratings as predictors of articles quality in Arts, Humanities and Social Sciences: an analysis based on the Italian Research Evaluation Exercise

**DOI:** 10.12688/f1000research.6478.1

**Published:** 2015-07-07

**Authors:** Andrea Bonaccorsi, Tindaro Cicero, Antonio Ferrara, Marco Malgarini

**Affiliations:** 1 ANVUR, Via Ippolito Nievo 35, Rome, 00153, Italy

**Keywords:** Journal rankings, ANVUR, Arts, Humanities and Social Sciences, Journal ratings, Article Quality

## Abstract

The aim of this paper is to understand whether the probability of receiving positive peer reviews is influenced by having published in an independently assessed, high-ranking journal: we eventually interpret a positive relationship among peer evaluation and journal ranking as evidence that journal ratings are good predictors of article quality. The analysis is based on a large dataset of over 11,500 research articles published in Italy in the period 2004-2010 in the areas of architecture, arts and humanities, history and philosophy, law, sociology and political sciences. These articles received a score by a large number of externally appointed referees in the context of the Italian research assessment exercise (VQR); similarly, journal scores were assigned in a panel-based independent assessment, which involved all academic journals in which Italian scholars have published, carried out under a different procedure. The score of an article is compared with that of the journal it is published in: more specifically, we first estimate an ordered probit model, assessing the probability for a paper of receiving a higher score, the higher the score of the journal; in a second step, we concentrate on the top papers, evaluating the probability of a paper receiving an excellent score having been published in a top-rated journal. In doing so, we control for a number of characteristics of the paper and its author, including the language of publication, the scientific field and its size, the age of the author and the academic status. We add to the literature on journal classification by providing for the first time a large scale test of the robustness of expert-based classification.

## Introduction

There is a large degree of agreement on the notion that research assessment in humanities and social sciences (HSS) is made more complex by a variety of factors. First, in these fields the structure of academic publication is largely different, with a large weight assigned to books and monographs, and to production in national language ( Finkenstaedt, 1990). Consequently, the bibliometric approach is considered to be of limited usefulness ( Nederhof *et al.*, 1989), not only because journals are a small fraction of total production and indexed journals are a tiny fraction of the population of journals in HSS, but also because the meaning of citations is different ( Frost, 1979). Third, and even more challenging, there is evidence that the number of research quality criteria is larger in HSS than in other fields, and also that there is less agreement on these criteria ( Hemlin, 1996; Hemlin & Gustafsson, 1996; Hug *et al.*, 2013; Hug *et al.*, 2014; Ochsner *et al.*, 2012; Ochsner *et al.*, 2013).

Faced with these challenges, the state of the art of the assessment of research in HSS at international level has followed several directions. On the one hand, it is agreed that peer review is still the most important evaluation methodology, so large efforts are made in making it more sophisticated, methodologically controlled, based on sound principles of evaluation methodology in social sciences, and free from unwanted biases, distortions and unexpected side effects. Under this agenda, issues such as the notion of originality, unorthodox science, or interdisciplinarity are under examination ( Guetzkow *et al.*, 2004; Hammarfelt, 2011). On the other hand, there are many efforts to classify and evaluate non-indexed journals (mainly in national languages), as one of the main vehicles for academic communication. An additional line of work refers to the classification of books and publishers.

This paper reports on a large experiment in the classification of journals in HSS carried out in Italy in the 2012–2014 period for the National Scientific Habilitation (Abilitazione Scientifica Nazionale; ASN). The exercise was based upon a mandatory provision in the law to rate *all* journals, in order to calculate the overall academic production of all candidates to the national procedure to become associate professor or full professor. This exercise asked the National Agency for the Evaluation of Universities and Research Institutes (ANVUR) to evaluate all journals in which at least one Italian scholar published at least one paper in the 2002–2012 period, for a total of more than 60,000 titles.

While the rating of journals has been followed in several national contexts, it is only in the Italian exercise that there is the opportunity to carry out a controlled experiment in order to test the robustness of journal classification. In fact, we have two independent evaluations carried out on the same set of journals. On the one hand, a panel of experts classified all journals as academic and non-academic (i.e. popular, professional, technical, cultural and political etc.), and rated the subset of academic journals in A-rated and non A-rated. The rating exercise was done on the basis of the reputation, esteem, diffusion and impact of journals, that is, on a qualitative, expert-based, reputational basis. On the other hand, we also have the rating of individual articles published in those journals, which have been done by a large number of individual referees (not panels) and summarized with a consensus agreement approach by expert panels, who however acted independently from the other panels, and without exchange of information. This peculiarity of the Italian context and the time sequence of events creates a favorable condition for carrying out a controlled experiment.

This paper extends to all HSS, with the exception of economics and business, the analysis initiated by Ferrara & Bonaccorsi (2015) on journals in the area of philosophy and history. In the following, we first introduce the database used for the analysis and hence we test for the influence of the journal class on the article score. Some consideration on the results obtained will conclude the paper.

## Methods

The paper is based on a dataset including data on all the journal articles submitted for evaluation by Italian scholars in the disciplinary areas of architecture, arts and humanities, history and philosophy, law and sociology and political science. Submissions for evaluation took place within the framework of VQR 2004–10, Italy’s national research assessment exercise involving all professors and researchers affiliated to the Italian universities and Public Research Organizations (PROs) as of November 2011. According to adopted rules, research evaluation in HSS was entirely based on peer review; research quality was assessed against the criteria of *relevance*, meaning contribution to the advancement of the state of art in the field, also in terms of adequacy, efficacy, timeliness and duration of impacts; *originality and innovation*, meaning contribution to the creation of new knowledge in the field; *internationalization*, meaning position in the international research landscape. Evaluation has been conducted by five Groups of Evaluation Experts (GEV in the Italian acronym), one for each area in HSS (Architecture; Arts and Humanities; History and Philosophy; Law; Sociology and political sciences); reviewers were instructed by GEV to evaluate articles only on the basis or their merit, regardless of the journal in which they are published in and of the language of publication. Each article had a possible rating of Excellent (A), Good (B), Fair (C) or Limited (D); to each class corresponded a score ranging from 1 (for articles A-rated) to zero (for articles deemed as limited). Negative scores were also assigned in case the article was deemed as non-academic (-1) or for plagiarism or fraud (-2, see Ancaiani *et al.*, 2015 for details). Limited to the human and social sciences, a substantial fraction of articles – namely, 6,701 out of 11,660 ( Table 1) – appeared on journals deemed as ‘A-class’ according to the procedure of ASN, intended to select the best researchers for the ranks of associate and full professors. Those journals, according to the relevant Ministerial Decree (No. 76/2012), were those ‘internationally recognized as excellent because of the rigor of their procedures of peer review and because of their diffusion among, esteem by, and impact on, the scholarly community of a field, as indicated also by their presence in the major national and international databases’ (our translation). Most of the remaining articles appeared on journals deemed as ‘academic’ for the purposes of the ASN, while a minority were published in journals that remained ‘uncategorized’. The main feature of the dataset, thus, is that it allows the comparison between the evaluations of journals and individual articles.

**Table 1.** Description of dataset.

Area of assessment	Acronym	Full Professor	Associate Professor	Researcher	Other	N° of articles	N° of articles in Class A journals
Architecture	Area08	280	278	353	7	918	360
Antiquities, philology, literary studies, art history	Area10	1,040	1,191	1,322	26	3,579	1,954
History, philosophy, pedagogy and psychology	Area11	713	680	726	8	2,127	1,086
Law	Area12	1,488	983	1,337	30	3,838	2,637
Political and social sciences	Area14	338	409	442	9	1,198	664
Total		3,859	3,541	4,180	80	11,660	6,701

A preliminary analysis shows that there is a relationship between the evaluation of individual articles and that of journals where the article is published ( Table 2). The non-parametric test for categorical data (Pearson χ ^2^) is statistically significant at 1% (All the statistical analyses have been performed using the software STATA ver. 13 ( http://www.stata.com/stata13/)), showing that the two distributions are not independent and hence the two ratings are mutually related. In the following, we will analyze more thoroughly this relationship, also controlling for a number of author-level and article-level variables.

**Table 2.** Preliminary analysis of association between the evaluation of research product and the evaluation of journal.

		Evaluation of journal			
		A	Not A	Not academic	Total
Evaluation of research product	A	1,344	573	20	1,937
	B	3,184	1,743	92	5,019
	C	1,322	1,096	80	2,498
	D	837	1,176	150	2,163
	Non-academic and other	14	21	8	43
	Total	6,701	4,609	350	11,660

*Pearson* χ ^2^ *=630.9; p-value=0.000*

## The influence of journal classification on the article score

We assume that the probability for an article i, published in the journal j, of receiving a score equal to *x* ∈ {-2; 1} is influenced by the class assigned to the journal, once controlling for a number of characteristics of the article:

*P* ( *Score _i,j_* = *x*) = *F*( *Journal class _i,j_, Paper characteristics _i,j_*)       (1)

Among the controls, we consider the language of publication (Italian or not) and the age (distinguishing among 3 age classes, less than 40 years, between 41 and 55 years and more than 55 years), scientific sector of activity (Scientific Areas 8, 10, 11, 12, 14), academic status (full professor; associate professor; researcher; other) and gender of the researcher. We also add the consideration of two binary variables controlling for the existence of international co-author(s) and for the nationality of the referees (allowing for the possibility of international referees). We finally add a variable taking into account the size of the scientific area of the author. The model is estimated as an ordered probit, an extension of the standard binary probit model, used when the dependent variable takes the form of a ranked and multiple discrete variable, considering alternatively the whole sample or each scientific area; in the first case, we also control for possible area-specific effects. In order to avoid the “dummy trap”, we normalize with respect to articles written in Italian with no international co-author, evaluated by an Italian reviewer, presented by a female researcher in sociology and political science, aged less than 40: i.e. the statistical significance, sign and magnitude of estimated parameters are to be interpreted as differentials with respect to this control group. The total number of available observations amounts to 11,660 varying from a minimum of 918 in architecture to a maximum of 3,838 in law ( Table 3).

**Table 3.** Ordered probit model (Dependent variable: article score).

Variables	Total	Architecture	Arts, & Hum.	Hist. & Phil.	Law	Sociology & Pol. Sci.
Journal rating	0.417***	0.542***	0.400***	0.379***	0.503***	0.323***
Architecture	0.134***					
Arts and Humanities	0.720***					
Hist. & Philosophy	0.471***					
Law	0.259***					
Italian language	-0.372***	-0.518***	-0.148***	-0.623***	-0.281***	-0.704***
41–55 years	-0.151***	-0.256	-0.208***	0.0492	-0.265***	-0.265**
More than 55 years	-0.582***	-0.662***	-0.726***	-0.394***	-0.563***	-0.572***
Associate professor	0.318***	0.206**	0.308***	0.265***	0.439***	0.234***
Full professor	0.818***	0.788***	0.690***	0.660***	1.096***	0.679***
Other personnel	-0.277**	-0.157	-0.329	0.359	-0.415**	-0.742*
Male	0.0777***	0.184**	0.0882**	0.0257	0.0542	-0.00491
International coauthors	0.301***	0.505***	0.122	0.237	0.902***	0.205
International reviewer	0.153***	0.363***	0.185***	0.0243	0.225***	0.0722
Number of Professor in the scientific sector (SSD)	-0.00131***	-0.00297***	-0.00248***	-0.00146**	-0.000921***	-0.00390***
Constant cut1	-2.854***	-2.514***	-2.081***	-2.990***	-1.867***	-2.953***
Constant cut2	-1.718***	0.363	-0.454***	-2.106***	-1.777***	-0.490***
Constant cut3	-1.695***	1.076***	0.200*	-0.357**	0.378***	0.402**
Constant cut4	0.302***	2.387***	1.574***	0.318**	1.162***	1.539***
Constant cut5	1.026***			1.651***	2.710***	
Constant cut6	2.400***					
Observations	11,660	918	3,579	2,127	3,838	1,198
Pseudo R-squared	0.0814	0.0919	0.0543	0.0720	0.0926	0.0832

*** p<0.01, ** p<0.05, * p<0.1

The main result is that both at the aggregate level and in each scientific area the article score is higher as the journal ranking gets better: in other words, the probability of receiving a high score grows if the article is published in a high-ranking journal according to the evaluation of the ASN’s experts. As for the control variables, we confirm most of the results already emerged in a previous paper on the same data ( Cicero *et al.*, 2014), namely, that article scores are higher for papers not written in Italian, with international co-authors, published by an under-40, male full or associate professor. Moreover, we also find that at aggregate level and in most areas an international reviewer and a lower number of professors in the specific scientific sector (SSD) are associated with an higher article score: a possible interpretation of the first result is that the expert groups responsible for the evaluation (GEV) mostly assign to international reviewers more internationalized papers, that are considered to have an higher probability of receiving a high score, given also that the level of internationalization was one of the evaluation criteria according to VQR rules (see again Ancaiani *et al.*, 2015). As for a negative relationship among area size and article score, this result emerged already in Ferrara & Bonaccorsi (2015) for the scientific fields in history and philosophy and is now extended to all HSS: a possible interpretation is that small fields may be favored by a “proximity bias” among authors and reviewers, thus resulting, *ceteris paribus*, in higher article scores.

As a final check, we concentrate on the probability of receiving an excellent score and relate it to the fact that the article is published in a top, A-Class journal, once controlling for the same variables considered in model 1:

*P* ( *Score _i,j_* = “ *E*”) = *F*( *Journal class _i,j_* = “ *A*”, *Paper characteristics _i,j_*)       (2)

In (2), F is the logistic function and the model is estimated as a logit, a class of models allowing to predict the binary response based on the specified predictors. A desirable feature of the logit model is that the regression coefficients may easily be transformed in odds ratio, expressing the change in the odds of the occurrence under scrutiny (in our case, the odds for a paper of receiving an ‘Excellent’ evaluation) due to a small change of a given predictor: in our case, we are particularly interested in the odds associated with the classification of a journal as a top, Class A journal. Estimation results for both the aggregate sample and each scientific area are presented in Table 4.

**Table 4.** Logit model (Odds ratio).

Variables	Total	Architecture	Arts, & Hum.	Hist. & Phil.	Law	Sociology & Pol. Sci.
Top Journal Classification	1.952***	2.513***	1.834***	2.424***	1.990***	1.311
Architecture	1.210					
Arts and Humanities	3.042***					
History and Philosophy	2.031***					
Law	1.084					
Italian language	0.488***	0.311***	0.681***	0.333***	0.529***	0.243***
41–55 years	0.878	0.408**	0.697**	1.144	0.807	0.671
More than 55 years	0.411***	0.248***	0.303***	0.506**	0.572***	0.252***
Associate professor	1.793***	1.283	1.815***	1.825***	2.629***	1.620*
Full professor	4.650***	3.263***	3.831***	4.023***	9.909***	4.877***
Other personnel	1.660	-	1.360	3.057	2.470	1.293
Male	1.247***	1.664**	1.263***	1.155	1.077	1.028
International coauthors	1.611***	2.357**	1.118	1.558	5.149***	1.511
International reviewer	1.352***	1.566**	1.393***	1.178	1.560***	1.490**
Full prof. in SSD	0.998**	0.992**	0.996**	0.998	1.000	0.990***
Constant	0.065***	0.201***	0.258***	0.137***	0.0332***	0.236***
Observations	11,660	911	3,579	2,127	3,838	1,198
Pseudo R-squared	0.116	0.140	0.0738	0.122	0.129	0.143

*** p<0.01, ** p<0.05, * p<0.1

According to logit estimations, the probability of receiving an excellent evaluation is positively affected by the journal in which the paper is published in: more specifically, publishing in a class A journal almost doubles the probability of receiving an excellent evaluation. Looking at the results in each scientific area, the odds of receiving an excellent evaluation are more than doubled by the publication in a Class A journal in architecture and history and philosophy; the effect is somewhat lower, but still highly significant, in law, and arts and humanities, while disappearing in sociology and political sciences. Logit estimation also broadly confirms the results already emerging from the ordered probit model: the odds of receiving an excellent evaluation are increased by publishing in a foreign language, with an international co-author (albeit only in law and architecture) and when the submitting author is 40 years old or younger, an associate or full professor and a male. Gender effect is in fact significant at the aggregate level and in architecture and humanities, but not in the remaining areas. Also in this case, having an international reviewer and publishing in a SSD characterized by a lower number of full professors helps in obtaining an excellent evaluation.

## Conclusions

Using a very large dataset of journal articles published in HSS, the paper proves that independent classifications of journals may be considered as good predictors of the score assigned to individual articles. More specifically, we find that, after controlling for a number of articles’ characteristics, the probability of receiving a better score grows with the quality profile of the journal the article is published in; moreover, the probability of receiving an excellent score almost doubles when the paper is published in a top, A-Class journal. The findings hold both at the aggregate level and for each specific sub-area considered in the analysis. While peer review has to remain the main evaluation methodology, our results indicate that journal classifications may be considered as a useful supporting tool in large evaluation exercise, since it may provide reviewers with valuable information apt to support expert evaluation.

## Data availability

The authors hold the view that it is important to allow the free access to data used in the article in order to enable others to replicate the study. However, information used in the article were gathered by the national agency responsible for evaluation of the University and research system in Italy (ANVUR), in the framework of this VQR exercise. In this context, ANVUR asked Italian professors to provide access to their publications, assuming the commitment not to disclose to the public, unless in an aggregate form, any data concerning the publications submitted for the evaluation and, most importantly, the results of the evaluation itself. This is deemed as necessary in order to guarantee the full anonymity of evaluations performed on each individual publications and on each Italian professor. For this reason, as the public agency in charge of evaluating research of Italian universities, ANVUR does not allow to make information about individual evaluations available to the general public.

The information used to generate data in this article concerning journal classification is available to the public at the following URL: http://www.anvur.it/index.php?option=com_content&view=article&id=254&Itemid=315&lang=it.
