# Supplementary material for: Journal ratings as predictors of articles quality in Arts, Humanities and Social Sciences: an analysis based on the Italian Research Evaluation Exercise
Source: F1000Res. 2015 Jul 7;4:196. [Version 1] doi: 10.12688/f1000research.6478.1 (PMC4536613; doi:10.12688/f1000research.6478.1)
Supplement: Peer review history

# Peer review: Referee response for version 1

**DOI:** 10.5256/f1000research.6951.r9400

**Published:** 2015-08-10

**Authors:** Chiara Faggiolani, Domenica Fioredistella Iezzi

**Affiliations:** 1 Science, Technology and Society, Università degli Studi di Roma , Rome, Italy; 2 Dipartimento di Ricerca Filosofica, Università degli Studi di Roma Tor Vergata, Rome, Italy

**Consistency of title and abstract and potential interest of the subject for readers**

The paper deals with a particularly relevant and controversial topic: journal ratings as predictors of articles quality in no-bibliometrics areas (architecture, arts and humanities, history and philosophy, law and sociology and political science).

The aim of this paper is to understand whether the probability of receiving positive peer reviews is influenced by having published in an independently assessed, high-ranking journal.

The title is appropriate for the content of the article and the abstract represent a suitable summary of the work. The paper is interesting and provides a useful exposure to the objectives proposed. It is well structured and clear in every step, although the article is brief and therefore omits some very important details.

  

**Article content: Have the design, methods and analysis of the results from the study been explained and are they appropriate for the topic being studied?**

The paper analyses a large dataset composed of over 11,500 research articles published in Italy in the period 2004-2010 in the areas of architecture, arts and humanities, history and philosophy, law, sociology and political sciences (non-bibliometric areas).

The authors used an ordered profit model to understand whether the probability of receiving positive peer reviews is influenced by having published in an independently assessed, high-ranking journal. The results show that independent classifications of journals may be considered as good predictors of the score assigned to individual articles.

In the manuscript, the authors use a big dataset that represents a very important first evaluation of the Italian university research. The paper is remarkable and underlines how a journal of high quality can ensure an excellent rating for a researcher. The method is suitable to achieve the goals. However, it should be highlighted that:

1) in the same non-bibliometric area, but in different scientific fields, monographs or articles of books rather than articles of the journal could be important, then, also kind of publication could be affect the evaluation.

2) the method adopted is appropriated, but it could be relevant applied clustering techniques to detect the profiles of micro-sectors (scientific fields), and find clusters of scientific fields, which although belonging to different areas have similar;

3) the paper deserves a greater depth to allow you to specify in detail the analysis. In fact, the authors present the results at macro level, and not enter into the details of different scientific fields. It would be interesting to have a longer version of the paper.

**Conclusions: Are the conclusions sensible, balanced and justified on the basis of the results of the study?**

Using a very large dataset of journal articles published in HSS, the paper proves that journal ranking may be considered as a good predictors of scores assigned to individual articles.

The probability of receiving an excellent score almost doubles when the paper is published in a top journal.

The results indicate that journal classifications may be a useful supporting tool in large evaluation excercise since it may provide reviewers with valuable information apt to support expert evaluation.

It is very important to emphasize, in agreement with *The Leiden Manifesto for research metrics *that «the best decisions are taken by combining robust statistics with sensitivity to the aim and nature of the research that is evaluated. Both quantitative and qualitative evidence are needed; each is objective in its own way. Decision-making about science must be based on high-quality processes that are informed by the highest quality data» ( Hicks, *et al*., 2015).

We have read this submission. We believe that we have an appropriate level of expertise to confirm that it is of an acceptable scientific standard.

# Peer review: Referee response for version 1

**DOI:** 10.5256/f1000research.6951.r9401

**Published:** 2015-08-04

**Authors:** Alesia Zuccala

**Affiliations:** 1 Royal School of Library and Information Science, University of Copenhagen, Copenhagen, Denmark

Brief Overview:

This study is situated within a national research evaluation context in Italy (i.e., designed for the selection of the best candidates for the ranks of associate and full professor) and focuses on two distinct rating exercises, one for journals and one for individual research articles published in five fields of the Social Sciences and Humanities. The authors take advantage of a fortuitous data set, and use an ordered probit-model to compare the score given by expert peer reviewers to 11,500 research articles (i.e., Excellent-A; Good-B; Fair-C; Limited-D) and the rating of the journal (classified as A or non-A) in which the individual articles were published. For all papers, a series of additional variables are taken into account, including a) language of the article, b) the scientific area of the author, c) the author’s age, academic status and gender, and d) the inclusion of an international co-author. In terms of referees, an allowance is made for the possibility that he/she was international. The purpose of this controlled experiment was to test for the robustness of expert-based journal ratings by determining the probability of a paper receiving a high independent review score, where the journal in which it was published also received a high independent score.

Assessment:

I tend to agree with the first reviewer in that more background information is needed regarding the criteria behind the original two rating exercises, although I basically find that for a very short article, the statistical aspects of the methodology are quite thorough.

I have only one comment about the rating exercises. The article indicates that different panels or groups of individual experts were chosen: some were assigned to provide the journal classifications (but we do not know how many), and a separate group of others (i.e., one non-panel, plus a consensus panel) were asked to rate individual articles from the specific fields (also how many?). Neither group were said to have exchanged information; thus acted independently of one another. The peer reviewers of the individual articles were “instructed to evaluate articles only on the basis of their merit regardless of journal and of language of publication” (p. 3).  Here, I am curious specifically about how much information pertaining to the journal (e.g., header; footer; abstract; citation style; volume number) had been removed prior to the article evaluation/rating procedure? Were the reviewers in this experiment truly blind to the journal’s influence? In certain areas of the Humanities, where there are notably fewer A-class journals, perhaps the format of the article instantly gave away the type of journal in which it was published. This could mean that when an article had been rated as being “excellent”, the reviewer was actually making a simultaneous judgement on the journal as well. I would therefore like to assume that all of the articles peer-reviewed in this experiment were non-formatted pre-prints (?); otherwise, this could be a factor which is contributing to the mutually related ratings.

I have read this submission. I believe that I have an appropriate level of expertise to confirm that it is of an acceptable scientific standard.

# Peer review: Referee response for version 1

**DOI:** 10.5256/f1000research.6951.r9402

**Published:** 2015-07-20

**Authors:** Geoffrey Williams

**Affiliations:** 1 EvalHum Initiative and Université de Bretagne-Sud, Lorient, France

**Overview**

This papers sets out to exploit some of the massive amount of data collected during the Italian 2012-2014 National Scientific Habilitation (Abilitazione Scientifica Nazionale; ASN). The database created for this evaluation exercise listed the academic output of all Italian researchers applying to become associate or full professors within the Italian university of research institutes. Data was classified by output type – academic, non-academic, journal articles, books, monographs… - and also by the disciplinary section of the candidate. Interestingly, the collected data for article publications went through two independent review processes with a panel ranking journals as academic and non-academic, and giving different weighting to the academic series, and another panel carrying out peer review to look at the quality of the articles submitted.

This aim of the study described in this paper was to see if there is agreement between the rankings produced by the two panels that would allow journal rank to be a reasonable proxy for quality. It looks at a selected number of very different fields from the HSS, namely architecture, arts and humanities, history and philosophy, law and sociology and political science. The study proceeds by a statistical analysis of journals ranking and the classification of individual articles given by reviewers on a scale from A (excellent) to D (limited). It also takes into account a series of variables such as the language of production and field size as well as the discipline and age of the writer.

The results are clearly of great interest in developing new bibliometric tools for handling data in large evaluation exercises in that they show a clear correlation between journal ranking and the outcome of peer review appraisal. The outcomes indicate that those articles with the best evaluation appeared in class A journals and that thus, although peer review remains important, a journal ranking may be a good proxy for quality.

**Assessment**

As this is a short article, much background knowledge would be needed to fully apprehend the criteria behind the original exercise. We have little information as to either the peer review panels and the reality of the criteria they applied, or how the panel classifying journals work, and the extent that it simply followed existing classifications as that of ERIH. These are both key elements in evaluating the output as they represent sources of potential bias and the influence of normative approaches to quality. The following comments thus concern more potential underlying biases in data collection and evaluation policy that the methodology applied here.

The first question applies to the ranking of the journals. Who are the experts and what is the danger of field bias? ERIH has come in for enormous criticism as the degree to which the experts represent a field is far from clear. In the case of this assessment, we do not know how the panels were constituted and to what extent the inclusion of a journal in an A list was free of domain bias. In France, the AERES agency had to abandon lists for the HSS as being too hotly disputed, and the field of law had simply ranked its own journals as A anyway. It is relatively easy to highlight a group of high profile, high impact journals in any field, but much more difficult to obtain clear criteria for ranking other journals as reputation measures can be fearfully biased.

The same problems arise with peer review, namely representativity of the panels and relevance of criteria to individual fields. We do not know how many reviewers read any individual paper and the extent they actually read the whole paper and were more competent to judge than the field specific peer reviewers who initially reviewed the article. The outcomes from the UK REF showed that reviewers only skimmed publications due to lack of time, given the volume of data to be treated, did these reviewers read more closely. Another issue arises from the allocation of experts, notably international experts who were chosen because the article was already deemed as having a quality potential.

Another important factor is the variety within the broad field of SSH, and even variety within disciplines. Architecture might be expected to have a more engineering dissemination profile, whereas political science and sociology can be at the end of the HSS spectrum that is closer to the sciences. The area 11, History, philosophy, pedagogy and psychology, is particularly wide as psychology can have a very different dissemination pattern to history, and is often not included or treated as borderline, within the HSS sphere. Within language, there will be great variation between areas. The oft-cited maxim that humanities researchers write books has been shown to be far too simplistic and is just an example of how broad brush strokes can hide the diversity within fields.

The article itself does point out that there can be perverse effects of an evaluation process, especially if it is normative. This may be happening here with internationalisation. Internationalisation is obviously an added value in research, but it should not be seen as a necessary prerequisite of quality. A relative lack of internationalisation is inherent in many humanities disciplines, and is particularly common in law. This is not a lack of quality, but simply due to the national orientation in the field of study. Penalising by too rigid evaluation criteria would be a bad thing as it is for evaluators to understand a field and adapt, not to try and change the field to suit their criteria.

These factors do not change the interest of the methodology adopted, but do need to be considered before making policy decisions based on outcomes. The methodology itself is through and opens vistas for analysing what is happening in assessment exercises and how it affects dissemination practice. I have only one minor gripe in what is otherwise a very clear and stimulating paper, and that is with data presentation. For the two datasets shown in tables 1 and 2, it would have been nice to have had averages shown so that we have an instantly visible means of comparison.  This would have revealed that law beats all disciplines with a very high level of A class journals, 68% as opposed to only 39% in architecture. These differences merit comment.

The article uses sound methodology and opens perspectives for more detailed work, and hopefully a close grain analysis within disciplines that will take into account researcher motivations. This is vital as correlation at this level does not necessarily justify the use of bibliometric criteria over peer review as there are inevitably built in biases in both quantitative and qualitative approaches. Until in-depth studies of how and why researchers disseminate are carried out, the picture will always be falsified. Thus, this research opens interesting channels, and always calls for much more sociological analysis before confirming outcomes.

I have read this submission. I believe that I have an appropriate level of expertise to confirm that it is of an acceptable scientific standard.
